# Application of a Solid Ceramic Membrane for Monitoring Volatile Organic Compounds in Industrial Wastewater

**DOI:** 10.3390/membranes10080186

**Published:** 2020-08-14

**Authors:** Injeong Kim, Jinseul Yoon, Sang Don Kim

**Affiliations:** 1 Jeonbuk Department of Inhalation Research, Korea Institute of Toxicology, Jeongeup 56212, Korea; injeongkim89@gmail.com; 2 School of Earth Sciences and Environmental Engineering, Gwangju Institute of Science and Technology, 123 Cheomdangwagi-ro, Buk-gu, Gwangju 61005, Korea; jinseulyun@gmail.com; 3 Center for Chemicals Risk Assessment, Gwangju Institute of Science and Technology, 123 Cheomdangwagi-ro, Buk-gu, Gwangju 61005, Korea

**Keywords:** volatile organic compound, passive sampling, solid ceramic dosimeter, HS-GC-MS, time-weighted average

## Abstract

A large quantity of volatile organic compounds (VOCs) can be released into water environments from oil spills and chemical exposure accidents. A recently developed solid ceramic dosimeter (SCD) could be used for long-term measuring of low VOCs concentrations in water. However, calibration and field testing of these SCDs have thus been far insufficient to apply for VOCs monitoring in a water environment in a chemical industrial area. We conducted laboratory calibration experiments and stability tests of the SCD. The mass accumulation of 14 target VOCs from 2 to 100 μg/L was increased linearly with time in the sampler. The absorption rate of the VOCs was related to Henry’s law constant. The average diffusion coefficient of the 14 VOCs in the SCD wall was 1.02 × 10^−9^ m^2^/s. The SCD was utilized in a petrochemical plant complex in South Korea with an industrial wastewater reservoir. After a total of 7 days of deployment, chloroform, ethylbenzene, and toluene were detected by both passive sampling and grab sampling at the same VOC concentrations.

## 1. Introduction

As the chemical industry in Korea has rapidly grown over the last several decades, the threat of chemical incidents has also been increasing. Between 1988 and 2006, more than 1000 chemical incidents occurred unofficially. Among them, 68% of incidents happened at fixed facilities, and petroleum fuel product-related incidents were the most frequent cases (235 cases) [1]. Chemical accidents result in environmental exposure of various toxic compounds, and adverse effects on the environment. Volatile organic compounds (VOCs) are low-molecular-weights substances, and accidents involving oil and organic solvents can release a large quantity of VOCs into the environment [2]. Although VOC concentrations in the water are not higher than in the air, they can last longer in water because of their low-to-medium water solubility [3]. Especially in the case of chemical accidents, the released VOC concentration in the water can be quite high—enough to induce acute toxicity to aquatic organisms. Long-term exposure to VOCs in water environments can cause carcinogenic effects, particularly in cold seasons [3,4]. To assess and manage the effects of chemical accidents, the quantification of even small amounts of VOCs is important. Because the effects of chemical accidents on human health and aquatic organisms can occur in both the short and long terms, continuous monitoring of VOCs in the water near chemical industrial areas is necessary.

Considering the low concentration of VOCs in water, and the need for long-term monitoring, passive sampling is the most appropriate method for VOC monitoring in water, rather than conventional grab sampling [5]. Grab sampling provides the contaminant concentration at only one point in time, and it can be useful to record episodic pollution for a short time. However, momentary concentrations of the contaminant can lead to an erroneous impact assessment if the contaminant has a long-term effect and fluctuates over time. Unlike grab sampling, passive sampling can measure the average concentration of the contaminant over time and the bioavailable concentration for organisms [5]. Passive sampling accumulates the analytes into the passive sampler at a particular site in the water. The accumulated analytes in the passive sampler are quite stable for a long time, which prevents their loss during transport and storage. In addition, passive sampling is easy, cost effective, and requires only simple extraction of the analytes from the sampler [6]. Due to these advantages, passive sampling is widely used for the measurement of hydrophobic organic contaminants in aquatic environments [7,8,9,10].

Among the types of passive samplers, ceramic dosimeters can be used to monitor VOCs in the water [11]. A ceramic dosimeter consists of two parts: the ceramic membrane tube and an absorbent. The ceramic membrane tube functions as a diffusion barrier that is water permeable, and the polymeric sorbent inside the ceramic membrane tube absorbs the contaminants. The type of sorbent is determined by the target analytes, and Dowex Optipore L-493 is used for VOC adsorption [12]. However, the performance of the water-permeable ceramic dosimeter can be affected by water viscosity and porosity [13]. Recently, a solid ceramic dosimeter (SCD) was developed for VOC passive sampling, which is made of a dense solid material and is impermeable to water. The SCD allows only gas-phase diffusion through the ceramic wall, so the Henry’s constant of the analytes determines the gas-phase diffusion in the SCD, not the water viscosity or porosity. The SCD showed a 60% higher average diffusivity of VOCs than a water-permeable porous ceramic wall, indicating more effective mass accumulation in the SCD [11]. In spite of the applicability of the SCD to groundwater monitoring, calibration and field testing of the SCD are necessary in order to apply it to VOC monitoring in the water near a chemical industrial area.

Use of the proper sampling and analytical methods for VOC monitoring in water is essential in order to evaluate the effect of VOCs that can be released due to chemical accidents. In the present study, 14 VOCs, including BTEX (benzene, toluene, ethylbenzene, and xylene), were selected as the target analytes. Our major objectives were to (1) validate the SCD passive sampler as an appropriate sampling method for these 14 VOCs in water and (2) assess the field application of this SCD to a wastewater reservoir in the petrochemical industry. The performance of passive sampling using this SCD was also be compared with that of conventional grab sampling.

## 2. Materials and Methods

### 2.1. Chemicals and Reagents

The properties of the target analytes (14 VOCs) are shown in Table 1. A standard solution mixture (Korean Drinking Water VOC Mix A) for the 14 VOCs, toluene-d8, chlorobenzene-d5, and dichloromethane (DCM) were obtained from Sigma Aldrich (St. Louis, MO, USA). Toluene-d8 and chlorobenzene-d5 were used as internal standards. DCM (≥99.5%) was used to extract the VOCs from the resin.

### 2.2. Preparation of the Solid Ceramic Dosimeter and Calibration

The solid ceramic dosimeter (COMA Technology, Gumi, Korea) consisted of a ceramic membrane tube, a Teflon cap, and a resin inside the tube. The length of the tube was 4 cm. The outer and inner diameter were 5 and 3 mm, respectively. The composition of the tube was 99.5% Al_2_O_3_, and its porosity was about 3% [11]. The Teflon cap did not affect the adsorption of VOCs, and prevented water from entering the tube. Before using the tube, the solid ceramic membrane tube was washed with soap and immersed in 1 M HCl (Sigma Aldrich, USA). Then, it was baked in an oven at 550 °C for 1 h. Dowex Optipore L493 (Sigma Aldrich, USA) weighing 0.14 g was added to each SCD. Dowex Optipore L493 is known as an adsorbent with a high accumulation rate and is suitable for VOC-adsorption [12]. Lastly, the prepared Teflon tube was sealed with the Teflon cap and Teflon tape to prevent entry of water.

Laboratory calibration of the SCD was performed for the selected 14 VOCs. A VOC mixture in methanol was spiked into deionized water at concentrations of 2, 10, 50, and 100 ng/mL at room temperature (≈20 °C). The total methanol concentration in each vial did not exceed 0.1%. All vials used in this experiment were washed with soap and subsequently washed with deionized water, and then placed in a furnace at 400 °C for 8 h to remove organics in the vials. The prepared ceramic dosimeters were placed in 40 mL vials, and the vials were filled with VOCs in water and kept at 50 rpm and 20 °C using a platform shaker. To maximize the salting out effect, 3.75 g of NaCl (Sigma Aldrich, USA) was added to each sample [11]. The SCD was taken out at each time interval (1–72 h) and the VOC concentrations in the water and resin were measured. For low concentration levels (2 ng/mL) of VOCs, a 250 mL vial was used. To extract the VOCs from the resin in the SCD, the resin (0.14 g per each SCD) was mixed with 1 mL of DCM in a 2 mL vial and agitated for 24 h by a rotator. VOCs in water were analyzed using HS-GC-MS, and the mass accumulations of VOCs in the resin were analyzed in the GC-MS.

### 2.3. Instrumental Analysis

The VOC concentrations of the samples were analyzed using gas chromatography-mass spectrometry (GC-MS) in the electron ionization mode. Chromatographic separation was performed on an Agilent 7890A GC system (Agilent Technologies, Santa Clara, CA, USA) equipped with an Agilent 5975C with a triple-axis detector (Agilent Technologies, USA). A split injection of 1 μL with a split ratio of 50:1 was made with the 7693A automatic sampler (Agilent Technologies, USA). A DB-624 UI capillary column (60 m × 250 μm × 1.4 μm, Agilent Technologies, USA) was used. Ultrahigh purity helium was used as the carrier gas, flowing at 1 mL/min. The oven was programmed at 40 °C for 5 min, 7 °C/min to 230 °C, and 230 °C for 5 min. The data were quantified using selective ion monitoring.

The extracted VOCs from the resin were analyzed using GC-MS, whereas Headspace-GC-MS (HS-GC-MS) was used to detect VOCs in the water samples. HS-GC-MS measurements were carried out with an HS autosampler (TriPlus 300, Thermo Scientific, Waltham, MA, USA). Table 2 shows the HS autosampler operating conditions. A 20 mL headspace screw vial (Thermo Scientific, USA) was used.

### 2.4. VOC Kinetics in the Solid Ceramic Dosimeter

**Figure 1:**
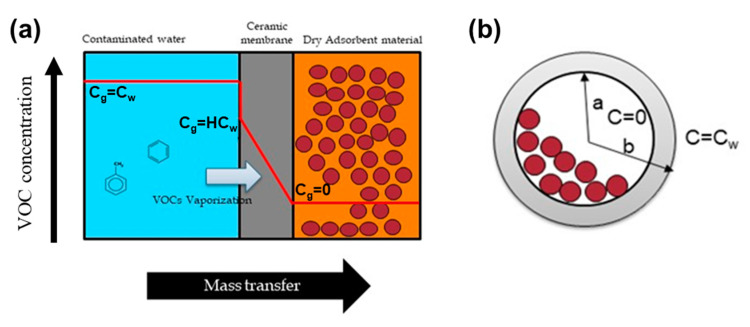
Concentration gradient profile between water and the solid ceramic membrane (**a**) and cross-section of the ceramic tube (**b**). The length of the tube (L) and inner (**a**) and outer (**b**) diameter were 40, 3, and 5 mm, respectively.

As shown in Figure 1a, VOCs in water are vaporized where contaminated water contacts the pores of the SCD. The vaporized VOCs pass through the pores of the ceramic membrane and adsorb to the adsorbent material in the tube. In other words, gas phase-VOCs diffuse through the solid ceramic wall and are being adsorbed to the resin. This ceramic membrane is in the shape of a tube, and it functions as a barrier that other chemicals with small Henry’s law constants cannot pass through (Figure 1b). The porosity of the solid ceramic tubes was low, at 3.3% [11]. Thus, liquid phase molecules cannot permeate into the ceramic tube.

A steady-state diffusion in the cylindrical tube can be expressed as:

(1) $$\frac{d}{dr}\left(r\frac{dC_{g}}{dr}\right)=0,\;\;a<r<b$$

where $C_{g}$ is the gas-phase vaporized VOC concentration in the membrane pores and a and b are the inner and outer radius, respectively. Boundary conditions of tube can be assumed below as:

(2) $$C_{g}=0\;at\;r=a,$$

(3) $$C_{g}=HC_{w}\;at\;r=b,$$

where $C_{w}$ is the aqueous-phase VOC concentration and H is the dimensionless Henry’s law constant (k_H_, Table 1). Therefore, $C_{g}$ can be solved as follows:

(4) $$C_{g}=\frac{HC_{w}\ln(r/a)}{\ln\left(b/a\right)},\;\;a<r<b$$

According to Fick’s first law, flux (J) in the tube can be described as:

(5) $$J=-D_{ceramic}\times \frac{dC_{g}}{dr}\;=\;\frac{H\cdot D_{ceramic}\cdot C_{w}}{\ln\left(b/a\right)\cdot r}$$

where $D_{ceramic}$ is the diffusion coefficient of the VOCs in the solid ceramic. The accumulated mass of the VOCs ($M_{t}$) through the ceramic tube can be derived as:

(6) $$M_{t}=J\times A\times t=\frac{2\pi HD_{ceramic}LC_{w}t}{\ln\left(b/a\right)}$$

where A is the surface area of the SCD, t is dosimeter deployment time in water, and L is the length of the SCD.

### 2.5. Field Application

The SCDs were deployed in a petrochemical plant complex reservoir in South Korea in April 2019. In the sampling area, there were a lot of petrochemical plants producing various aromatic compounds and synthetic resins through petrochemical processes, and a benzene spill occurred in the area in January 2018. The SCDs were stored in sealed plastic bags during transport and deployed with stainless cages to protect the samplers in the sampling site. They were placed 30 cm below the water surface using a buoy. The SCDs were taken out at 2, 5, and 7 days after installation. At each sampling time, grab sampling was also performed according to EPA method 5035A [18]. Industrial wastewater was collected using bailers, and we filled 40 mL amber vials with 2 mL of HCl (37%, Sigma Aldrich, USA) and 25 mg ascorbic acid (Sigma Aldrich, USA) to prevent dechlorination and biodegradation during transportation and storage. The collected samples were kept at a low temperature (≈4 °C) and transported to the laboratory within 6 h of sampling. VOCs in the resin of the SCDs were extracted, as described in Section 2.2, and analyzed by GC-MS. The water samples were filtered with 0.45 μm membrane filters, and VOC concentrations in the water were measured using HS-GC-MS.

## 3. Results and Discussion

### 3.1. Validation of the Analytical Method

The method detection limit (MDL), limit of quantification (LOQ), relative standard deviation (RSD), and recovery were calculated to measure the performance of the analytical method. The MDL is the lowest measured concentration of the compounds that enable them to be differentiated from the method blank with 99% confidence [19]. The MDL is obtained by the student’s *t* value for a 99% confidence interval for seven replicates (3.14). The LOQ is defined as the minimum concentration of the compounds that can be determined with an acceptable concentration level of precision and trueness. The LOQ is denoted as 10 times the standard deviation. This was calculated by analyzing the concentration of each VOC in the seven samples. Precision was indicated as the relative standard deviation. The analyzed results of the concentrations using the HS-GC-MS method for the MDL and LOQ were 0.13–1.03 and 0.40–3.29 μg/L, respectively (Table 3 and Figure S1). The results using the GC-MS method for the MDL and LOQ were 5.43–84.17 and 17.28–267.81 μg/L, respectively (Table S1). The recovery ranged from 70% to 120%, and the RSD values were below 20%. According to the European Commission SANTE/11813/2017 [20], the majority of VOCs showed an acceptable performance.

### 3.2. Calibration and Performance of the Solid Ceramic Dosimeter

**Figure 2:**
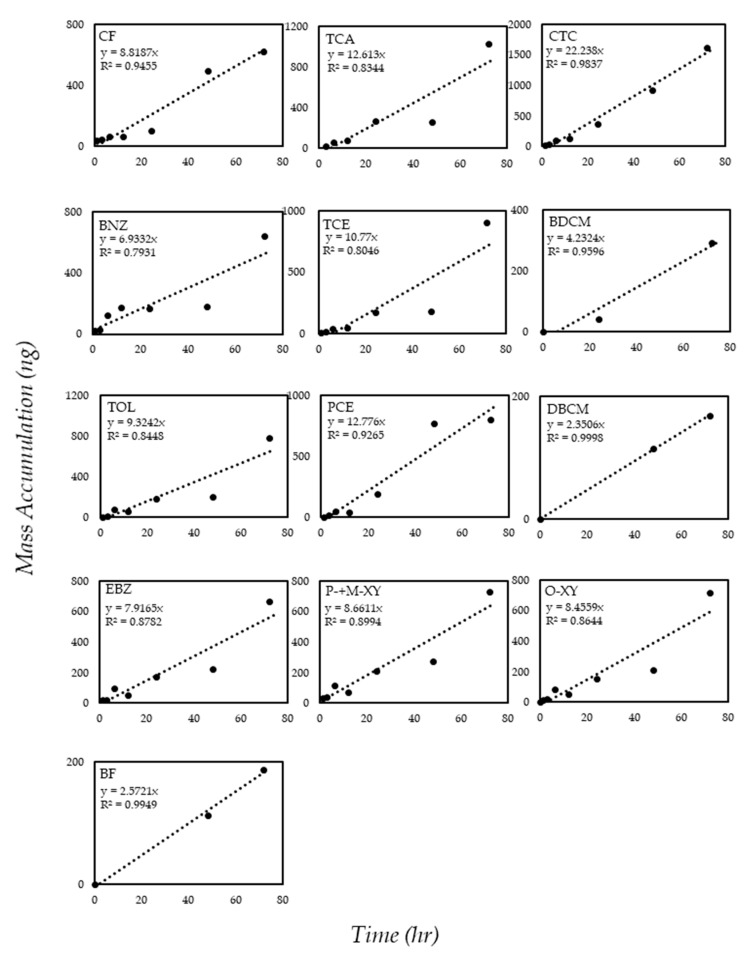
The mass accumulation of volatile organic compounds (VOCs) with time, representing the linear kinetics of the solid ceramic dosimeter at a VOC concentration of 43.11 (±6.72) μg/L.

In the laboratory calibration, we confirmed that the accumulated mass of the VOCs increased linearly over time, as shown in Figure 2. The VOC absorption showed good linearity in a broad range from 2 to 100 μg/L at room temperature (≈20 °C). This indicates that the VOCs were vaporized from water and were absorbed on the resin (Dowex Optipore L-493) of the SCD consistently. The spiked VOC concentrations in the water were 2, 10, 50, and 100 μg/L. The measured VOC concentrations in the water were 2.53 (±0.69), 8.21 (±1.13), 43.11 (±6.72), and 81.36 (±15.36) μg/L, respectively. The slopes of each line in Figure 2 are a function of D_ceramic_, H, a, b, L, and C_w_ (in Section 2.4), representing the linear kinetics of the SCD at a VOC concentration of 43.11 (±6.72) μg/L. The mass accumulations of VOCs over time by the SCD membrane at various concentrations are shown in Figures S2–S5. The maximum amount of chemical absorbed in the resin in this experiment was 18.4 μg in 0.14 g of Dowex Optipore resin, when adsorbed for the longest time (72 h) at 100 μg/L.

Bonifacio et al. [11] conducted the laboratory calibration of the SCD at VOC concentrations from 16.9 to 1100 μg/L. In addition, the linearity of the VOC absorption kinetics was decreased at low VOC concentrations. The VOC concentration for the laboratory calibration was much higher than that of this study. Since VOCs exist at a concentration much lower than 100 μg/L in the actual environment, the present study proved the linear absorption of VOCs by the SCD at environmentally relevant concentrations. Even though five VOCs were not able to be measured by absorption kinetics at the lowest concentration (2.53 μg/L), the linearity of the VOC absorption kinetics in this study was good at all concentrations. This indicates that the SCD used in this study has a good enough sorption capacity to detect VOCs in actual water environments.

Table 4 shows the D_ceramic_ values calculated from each point in this study and from the previous study [11]. At 2 μg/L, five VOCs could not be quantified, because the analysis result was below the LOQ concentration. Since these compounds have a low Henry’s law constant, a smaller amount of VOCs accumulated in the SCD. The D_ceramic_ in this study was 3 to 10 times higher, indicating a faster VOC uptake in our SCD than in the previous study [11]. The characteristics of the SCD used in the present study were the same as the previous one, except for the diameter of the ceramic tube. The outer diameter was 5 mm in this study, and 4 mm in the previous study. The surface area of the SCD was increased by about 25% in this study. As a diffusion barrier, the increased surface area of an SCD can result in a higher uptake rate of contaminants [9]. In addition, we increased the amount of resin in the SCD. Bonifacio et al. [11] used 0.08 g of resin, while we added 0.14 g of resin per SCD. The enhanced VOC sorption capacity, even at low concentrations, was achieved from these differences; that is, the increased surface area of the SCD and the increased amount of resin.

**Figure 3:**
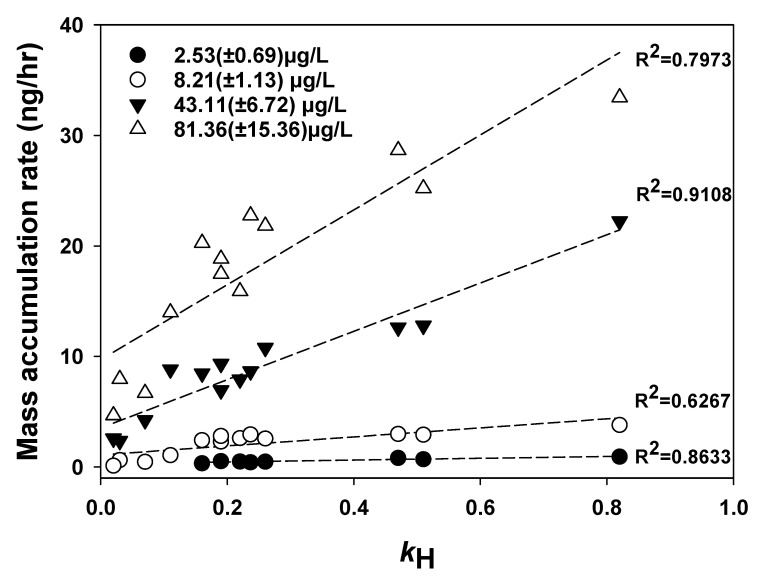
Correlation between the Henry’s law constant (k_H_ at 20 °C) and the VOC mass accumulation rate in the solid ceramic dosimeter.

In Figure 3, the correlation between the Henry’s constant and the mass accumulation rate of the SCD was obvious at concentrations ranging from 2 to 100 μg/L. Each circle in Figure 3 represents the slope of the graphs, showing the correlation of the mass accumulation with absorption time in Figure 2 and Figure S2–S5. The higher the dimensionless Henry’s law constant, the greater the vaporization of VOCs on the surface of the solid ceramic tube. Consequently, a larger concentration gradient occurs between the inside and outside of the tube, resulting in a large mass flux. Unlike in a porous ceramic dosimeter, this SCD showed very small porosity (about 3%), and was impermeable to water. Since the SCD absorbs VOCs only in the gas-phase, the VOC uptake of the SCD was not affected by the hydrodynamic flow and viscosity of the aqueous environment [11,13]. Thus, the Henry’s law constant of the compound is considered the most important determinant of compound uptake of this SCD.

### 3.3. Stability of the SCD at Low Temperatures

**Figure 4:**
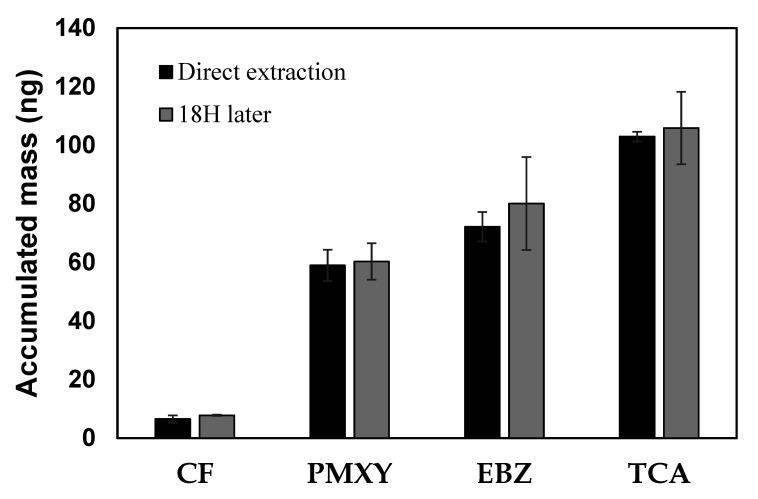
Accumulated mass in the resin of the solid ceramic dosimeter by direct extraction and extraction after 18 h. Error bars represent the standard deviation (n = 5).

Since the sample treatment could not be conducted right after retrieving the SCD from the sampling site, we checked the absorption stability of the VOCs in the SCD. Each of the 10 SCDs were placed in a solution containing 10 ng/mL of the 14 VOCs for 3 days. Half of the dosimeters were immediately extracted using dichloromethane. The other half were sealed using small zipper storage bags at a low temperature (<4 °C) for 18 h and then extracted. As shown in Figure 4, there was no significant difference in the accumulated masses of four VOCs in the Dowex resin between direct extraction and extraction after 18 h. The recovered masses of the other VOCs are not shown, but similar results were achieved. If not sealed, VOCs on the resin can come in contact with the air and be vaporized. However, VOCs in resin inside the SCD were not desorbed when it was blocked from air at a low temperature (<4 °C). It was confirmed that there are no losses of the adsorbed VOCs on the resin when the SCDs are sealed at low temperatures (<4 °C).

### 3.4. Field Application to Industrial Wastewater

To verify the applicability of the SCD to VOC monitoring in water, we measured the VOC concentrations in treated industrial wastewater using two different sampling methods: passive sampling using the SCDs and grab sampling. In a preliminary test, we conducted grab sampling at five points of a sampling site (A to E) in a petrochemical plant complex reservoir in South Korea in November 2018. In this sampling area, there were a lot of petrochemical plants producing various aromatic compounds and synthetic resins through petrochemical processes, and a benzene spill occurred in the area in January 2018. As shown in Table 5, eight VOCs were found at low concentrations (1.15–66.14 μg/L) at sampling sites A to E. The highest number of VOCs (eight VOCs) was detected at point D.

Based on the results shown in Table 5, the passive sampler was installed in sampling site D in April 2019 for 7 days. BNZ, BDCM, DBCM, PXY, MXY, OXY, and BF were detected, but they were lower than LOQ. Only CF, TOL, and EBZ were quantified. The lower VOC concentrations indicated by passive sampling than those in the preliminary test probably resulted from seasonal differences between the two sampling periods. The water temperature in April is higher than in November, and VOCs easily evaporate from the water at a high water temperature.

**Figure 5:**
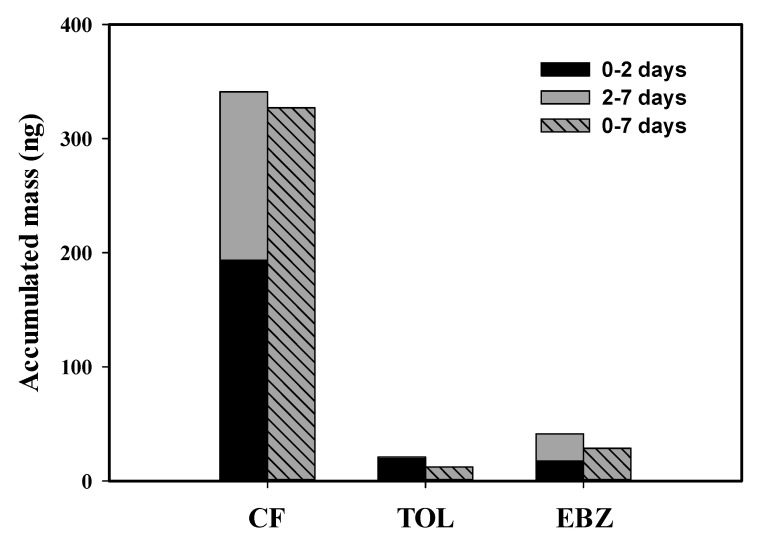
Accumulated masses of three VOCs on the solid ceramic dosimeter in the treated industrial wastewater.

Figure 5 shows the three accumulated VOC masses of the SCD placed in the reservoir of treated industrial wastewater. Two sets of passive samplers, which were installed in different sampling periods, were deployed in the reservoir to determine the time-weighted VOC accumulation of samplers for 7 days. VOCs accumulated more in the passive sampler for the first 2 days (days 0–2) than in the last 5 days (days 2–7). However, a good accordance was found when the sum of the VOC masses absorbed in the dosimeter during each period (days 0–2 and 2–7) was compared to the mass analyzed after the entire deployment period (days 0–7). In other words, the SCD worked well in this wastewater deployment. A low accumulated mass in the sampler installed for days 2–7 represents a low concentration during that period. This was caused by seawater dilution in the reservoir due to opening of the gates between days 2 and 7. The accumulated VOC masses absorbed over the whole deployment period (days 0–7) was slightly lower than the total amount of accumulated masses in the dosimeter used in the first period (days 0–2) and the second period (days 2–7). The average concentrations of VOCs in the SCD samplers were constantly maintained at a steady level considering the dilution of the second sampling period on days 2–7. We observed that the surfaces of the dosimeters after 7 days were covered by contaminants. Fouling on the ceramic membrane wall can affect the accumulation of VOCs. In river water, the surface of the porous ceramic membrane was discolored after 1 week of deployment, as in our study, and the uptake of contaminants by the sampler was notably decreased after 3 weeks [9]. However, fouling of the porous ceramic dosimeter was not observed in groundwater for 1 year [8]. These findings indicate that the performance and applicability of the SCD in the field should be confirmed when it is intended to be installed for a longer period of time.

To evaluate the performance of the SCD for VOCs in industrial wastewater, the water concentrations derived from passive sampling were compared to the grab sampling water concentrations over 2 days. Table 6 represents a comparison of the VOC concentrations derived by the SCD (time-weighted average concentration) and grab sampling in the industrial wastewater reservoir. The water concentrations estimated from the SCD were obtained at an average water temperature of 23 °C. Results of *t*-test show that there is no statistically significant difference between the mean concentration estimated by grab sampling and the time-weighted average concentration obtained by passive sampling. Although there was no statistical difference between the two sampling methods, grab sampling showed the higher average concentration compared to passive sampling. Generally, particulate matter or colloid in the water can be collected by grab sampling, and it may cause a higher concentration of the contaminant to be measured compared to passive sampling if the contaminant exists in particulate matter or colloid. Unlike grab sampling, passive sampling uptakes only the dissolved contaminant in the water [8]. In addition, the standard deviation of grab sampling is larger than that of passive sampling. This indicates that passive sampling is a more stable sampling method to measure the average concentration of a contaminant. The water environment of our sampling site (e.g., wastewater reservoir) is frequently changed by an inflow of treated water and opening of the reservoir gate, which allows seawater into the reservoir. Passive sampling is better than grab sampling when there is a fluctuation in the water environment and when long-term effects should be considered.

## 4. Conclusions

This study evaluated the performance of a solid ceramic dosimeter as a passive sampler in industrial wastewater. All 14 VOCSs were adsorbed by the resin, and the mass accumulation rate was correlated with Henry’s law constant. The enhanced VOC sorption capacity, even at low concentrations, was achieved from the increased surface area of the SCD and the increased amount of resin. The results confirmed that there are no losses of the adsorbed VOCs on the resin when the SCDs are sealed at low temperatures (<4 °C). In field application, passive sampling using the SCD showed the same concentration levels as those obtained by the grab sampling method. Consequently, passive sampling using this SCD is an efficient tool for analyzing the time-weighted average concentration of VOCs. However, a long-term field test may result in contamination of the ceramic membrane walls, which could be a limiting factor for the use in industrial wastewater. Even though the accumulated mass of the contaminants is not reached to the maximum absorption capacity of SCD (more than 1 year), the fouling on the surface of SCD can reduce the accumulation rate of the contaminants on SCD. Therefore, in case of the long-term monitoring, the fouling effect of SCD should be considered.

## Figures and Tables

**Table 1:** Properties of the 14 volatile organic compounds (VOCs) [14,15,16,17].

Compound	Abbreviation	Predominant Use Group	CAS No.	Toxicity	Henry’s Law Constant (k_H_, at 283 K)
1,1,1-Trichloroethane	TCA	Solvent	71-55-6	Carcinogen	0.465
Carbon tetrachloride	CTC	Solvent	56-23-5	Carcinogen	0.822
Tetrachloroethylene	PCE	Solvent	127-18-4	Carcinogen	0.511
Trichloroethene	TCE	Solvent	79-01-6	Carcinogen	0.256
Bromodichloromethane	BDCM	Trihalomethane (chlorination by-product)	75-27-4	Carcinogen	0.074
Chloroform	CF	Trihalomethane (chlorination by-product)	67-66-3	Carcinogen	0.111
Bromoform	BF	Trihalomethane (chlorination by-product)	75-25-2	Carcinogen	0.017
Dibromochloromethane	DBCM	Trihalomethane (chlorination by-product)	124-48-1	Carcinogen	0.034
Benzene	BNZ	Gasoline hydrocarbon	71-43-2	Carcinogen	0.19
Toluene	TOL	Gasoline hydrocarbon	108-88-3	Carcinogen	0.186
Ethylbenzene	EBZ	Gasoline hydrocarbon	100-41-4	Carcinogen	0.218
p-Xylene	PXY	Gasoline hydrocarbon	106-42-3	Toxic	0.246
m-Xylene	MXY	Gasoline hydrocarbon	108-38-3	Toxic	0.226
o-Xylene	OXY	Gasoline hydrocarbon	95-47-6	Toxic	0.157

**Table 2:** Conditions of the Headspace autosampler.

Oven temperature	65 °C	Loop fill mode	Standard
Manifold temperature	260 °C	Loop fill time	2.5 min
Transfer line temperature	260 °C	Loop equilibrium time	2.5 min
Vial equilibrium time	25 min	Injection time	2 min
Shaking	Low	Aux flow	30 mL/min
Mode	Flow	Auxiliary gas	He
Aux pressure	100 kPa	Vial press	5 kPa
Aux flow	30 mL/min	Communication pipe	Nope
Pressure equilibrium time	1 min		

**Table 3:** Retention time (RT) and selected reaction monitoring transition of HS-GC-MS for 14 VOCs.

Compound	RT (min)	Primary Ion (Da)	HS-GC-MS				
			Spiking Level (ug/L)	MDL (ug/L)	LOQ (ug/L)	RSD (%)	Recovery (%)
CF	12.9	83	0.5	0.23	0.72	20.02	71.69
TCA	13.36	97	0.5	0.22	0.71	14.46	98.48
CTC	13.71	117	1	0.21	0.68	6.7	102.03
BNZ	14.07	78	2	1.03	3.29	13.92	118.15
TCE	15.32	95	0.5	0.24	0.76	13.32	114.45
BDCM	16.42	83	2	0.91	2.88	15.96	90.33
TOL	17.83	92	0.5	0.13	0.4	11.43	70.86
PCE	18.98	164	2	0.53	1.67	6.9	121.29
DBCM	19.42	129	2	0.68	2.18	12.31	88.3
EBZ	20.87	91	0.5	0.18	0.58	6.06	95.85
PXY and MXY	21.12	91	1	0.27	0.86	8.35	102.42
OXY	21.94	91	1	0.2	0.64	5.57	115.53
BF	22.39	173	2	0.81	2.58	14.86	86.88

**Table 4:** Average D_ceramic_ values at various concentrations in this study and in a previous study.

VOCs	D_ceramic_ (× 10^−9^ m^2^/s)						
	2.53 μg/L	8.21 μg/L	43.11 μg/L	82.08 μg/L	Avg.	SD	Bonifacio et al. [11]
CF		1.36	1.32	0.92	1.20	0.20	
TCA	0.79	0.63	0.47	0.63	0.63	0.11	0.20 (±0.02)
CTC	0.76	0.63	0.54	0.63	0.64	0.08	
BNZ		1.08	1.21	1.04	1.11	0.07	0.10 (±0.03)
TCE	0.8	1.12	0.56	1.17	0.91	0.25	0.09 (±0.02)
BDCM		0.64	0.66	0.68	0.66	0.02	
TOL	1.93	1.31	0.91	1.12	1.32	0.38	0.13 (±0.002)
PCE	0.81	0.75	0.61	0.62	0.70	0.09	0.09 (±0.01)
DBCM		1.73	1.36	1.22	1.44	0.22	
EBZ	1.87	1.34	0.95	1.02	1.30	0.36	0.13 (±0.03)
PXY + MXY	1.49	1.59	1.32	0.85	1.31	0.28	0.12 (±0.01)
OXY	1.38	1.46	1.04	1.18	1.27	0.17	
BF		0.59	0.87	0.84	0.77	0.13	

**Table 5:** VOC concentrations (μg/L) in the wastewater reservoir of a petrochemical plant complex in South Korea.

Compound	Point A	Point B	Point C	Point D	Point E
CF	<LOQ	<LOQ	<LOQ	2.00	<LOQ
TCA	ND	ND	ND	ND	ND
CTC	ND	3.03	1.40	9.22	9.57
BNZ	<LOQ	<LOQ	<LOQ	<LOQ	<LOQ
TCE	ND	ND	ND	ND	ND
BDCM	<LOQ	<LOQ	ND	4.55	<LOQ
TOL	3.03	ND	<LOQ	1.15	ND
PCE	ND	ND	ND	ND	ND
DBCM	14.84	8.25	ND	6.10	ND
EBZ	4.38	1.73	1.62	2.99	1.38
PXY and MXY	5.12	4.75	4.81	4.72	4.58
OXY	1.92	1.77	1.82	ND	1.78
BF	66.14	32.46	ND	8.18	ND

**Table 6:** Comparison of the grab sampling and passive sampling methods for 2 days of collection. The *p*-values are from a *t*-test at α = 0.05.

Compound	Passive Sampling		Grab Sampling		*p*-Value
	Avg.	SD	Avg.	SD	
CF	13.2902	3.1152	20.8662	7.4238	0.51
TOL	0.5004	0.0781	0.9165	0.7056	0.82
EBZ	0.4542	0.0776	0.9242	0.0698	0.96

## Supplementary Materials

The following are available online at https://www.mdpi.com/2077-0375/10/8/186/s1, Figure S1: HS-GC/MS total ion chromatogram of VOCs at 200 μg/L, Figure S2: The mass accumulation of VOCs with time, representing the linear kinetics of the solid ceramic dosimeter at a VOC concentration of 2.53 (±0.69) μg/L, Figure S3: The mass accumulation of VOCs with time, representing the linear kinetics of the solid ceramic dosimeter at a VOC concentration of 8.21 (±1.13) μg/L, Figure S4: The mass accumulation of VOCs with time, representing the linear kinetics of the solid ceramic dosimeter at a VOC concentration of 43.11 (±6.72) μg/L, Figure S5: The mass accumulation of VOCs with time, representing the linear kinetics of 81.36 (±15.36) μg/L, Table S1: Retention time (RT) and SRM transition for VOCs of GC-MS.
