# Supplementary material for: Application of a Solid Ceramic Membrane for Monitoring Volatile Organic Compounds in Industrial Wastewater
Source: Membranes (Basel). 2020 Aug 14;10(8):186. doi: 10.3390/membranes10080186 (PMC7465513; doi:10.3390/membranes10080186)
Supplement: Supplementary file 1 [file membranes-10-00186-s001.pdf]

membranes

Supporting Information: Application of a Solid Ceramic Membrane for Monitoring Volatile Organic Compounds in Industrial Wastewater

Injeong Kim [1,†], Jinseul Yoon [2,†] and Sang Don Kim [2,3,*]

1 Jeonbuk Department of Inhalation Research, Korea Institute of Toxicology, Jeongeup, 56212, Korea; injeongkim89@gmail.com
2 School of Earth Sciences and Environmental Engineering, Gwangju Institute of Science and Technology, 123 Cheomdangwagi-ro, Buk-gu, Gwangju, 61005, Korea; jinseulyun@gmail.com
3 Center for Chemicals Risk Assessment, Gwangju Institute of Science and Technology, 123 Cheomdangwagi-ro, Buk-gu, Gwangju, 61005, Korea
† Authors 1 and 2 equally contributed as a first author
* Correspondence: sdkim@gist.ac.kr; Tel: +82-62-715-2445

Received: 27 July 2020; Accepted: 12 August 2020; Published: date

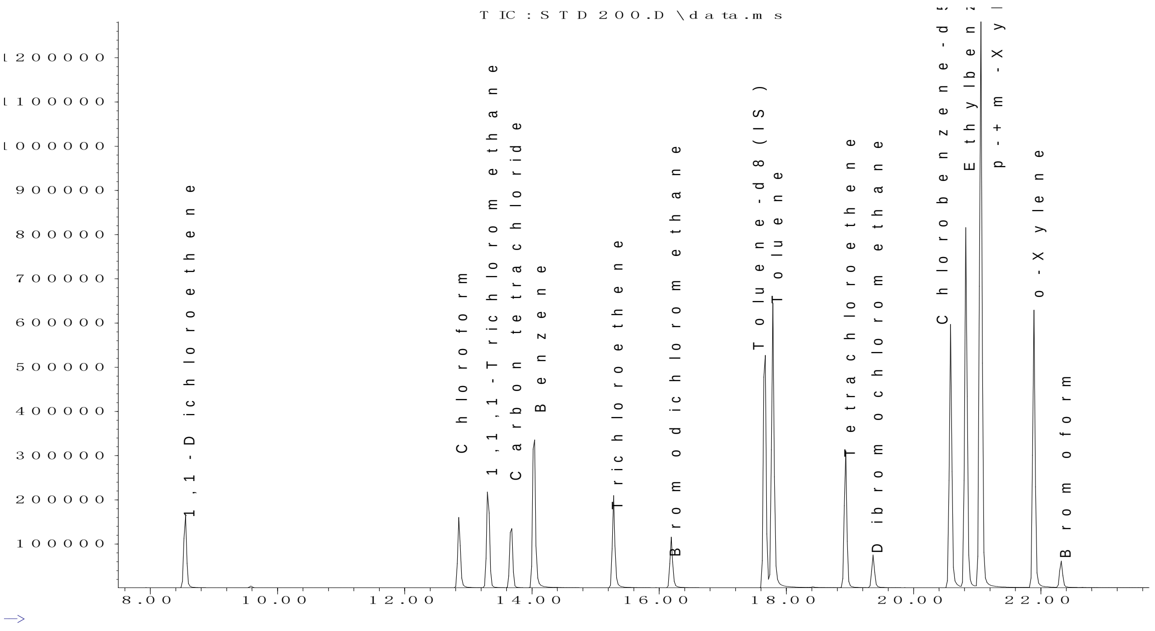

Figure S1. HS-GC/MS total ion chromatogram of VOCs at 200 μg/L.

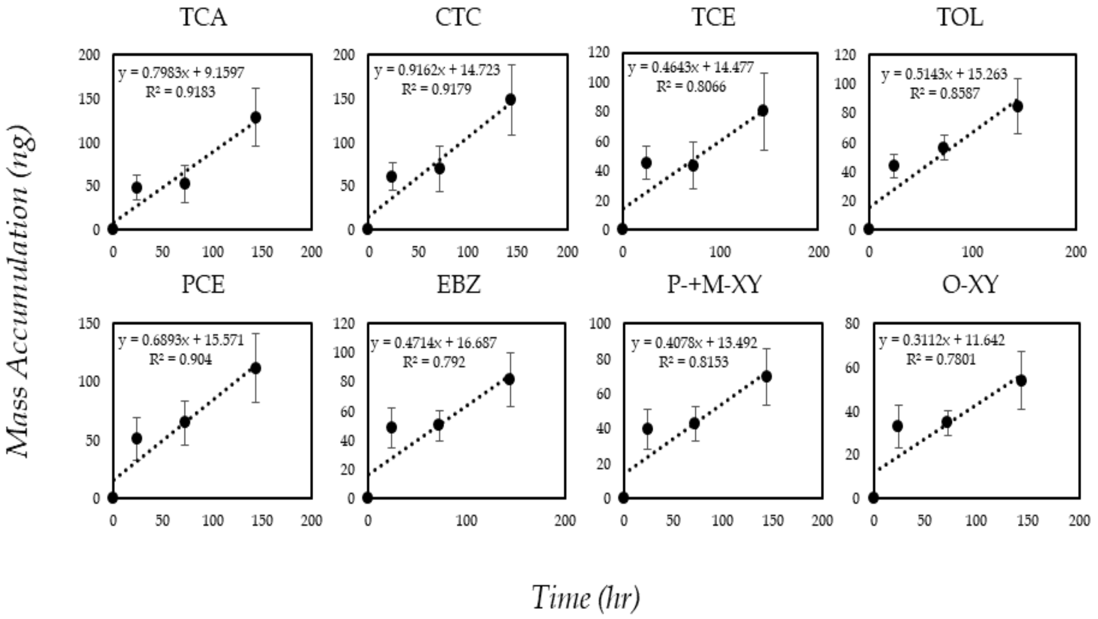

Figure S2. The mass accumulation of VOCs with time, representing the linear kinetics of the solid ceramic dosimeter at a VOC concentration of 2.53 (±0.69) μg/L.

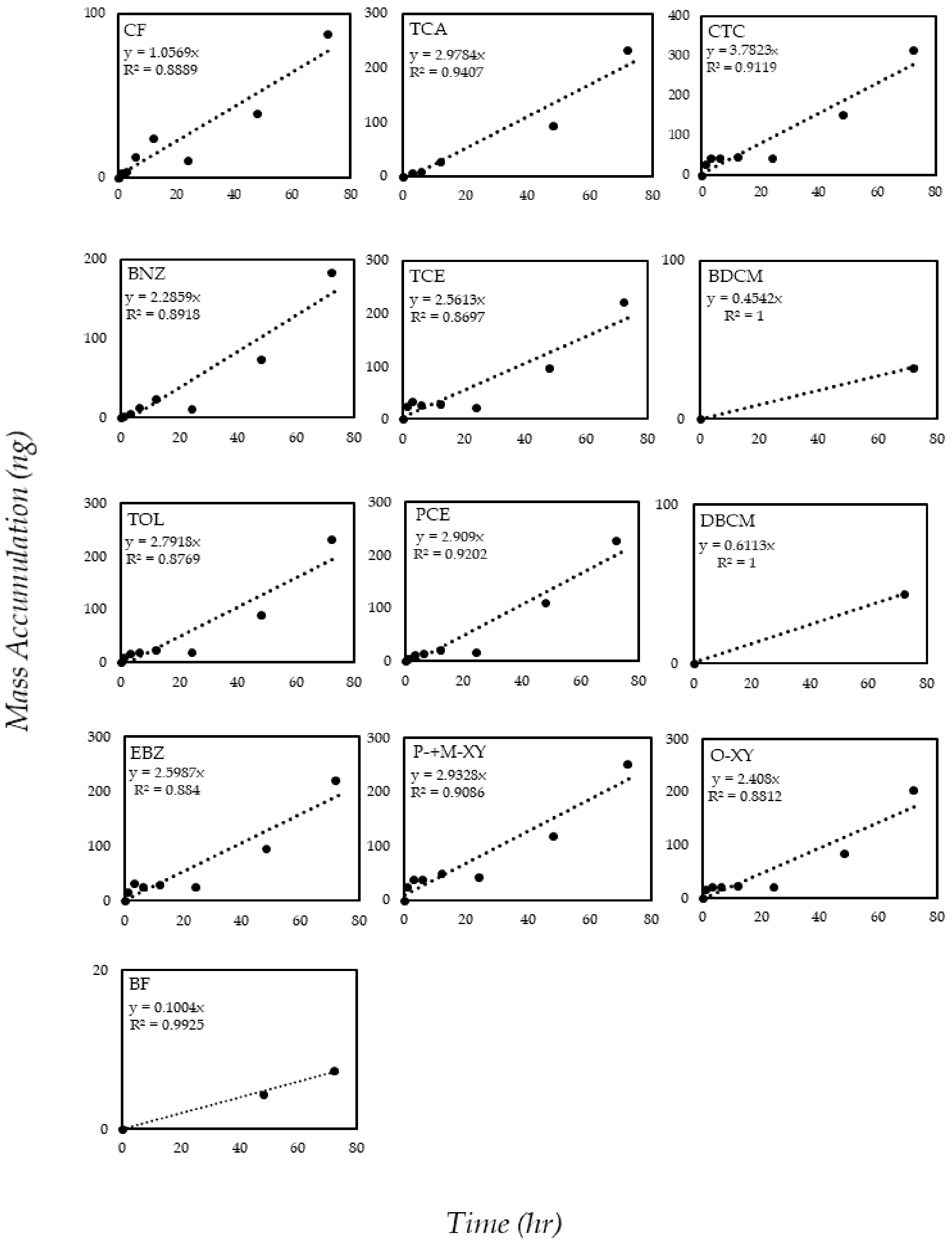

Figure S3. The mass accumulation of VOCs with time, representing the linear kinetics of the solid ceramic dosimeter at a VOC concentration of 8.21 (±1.13) μg/L.

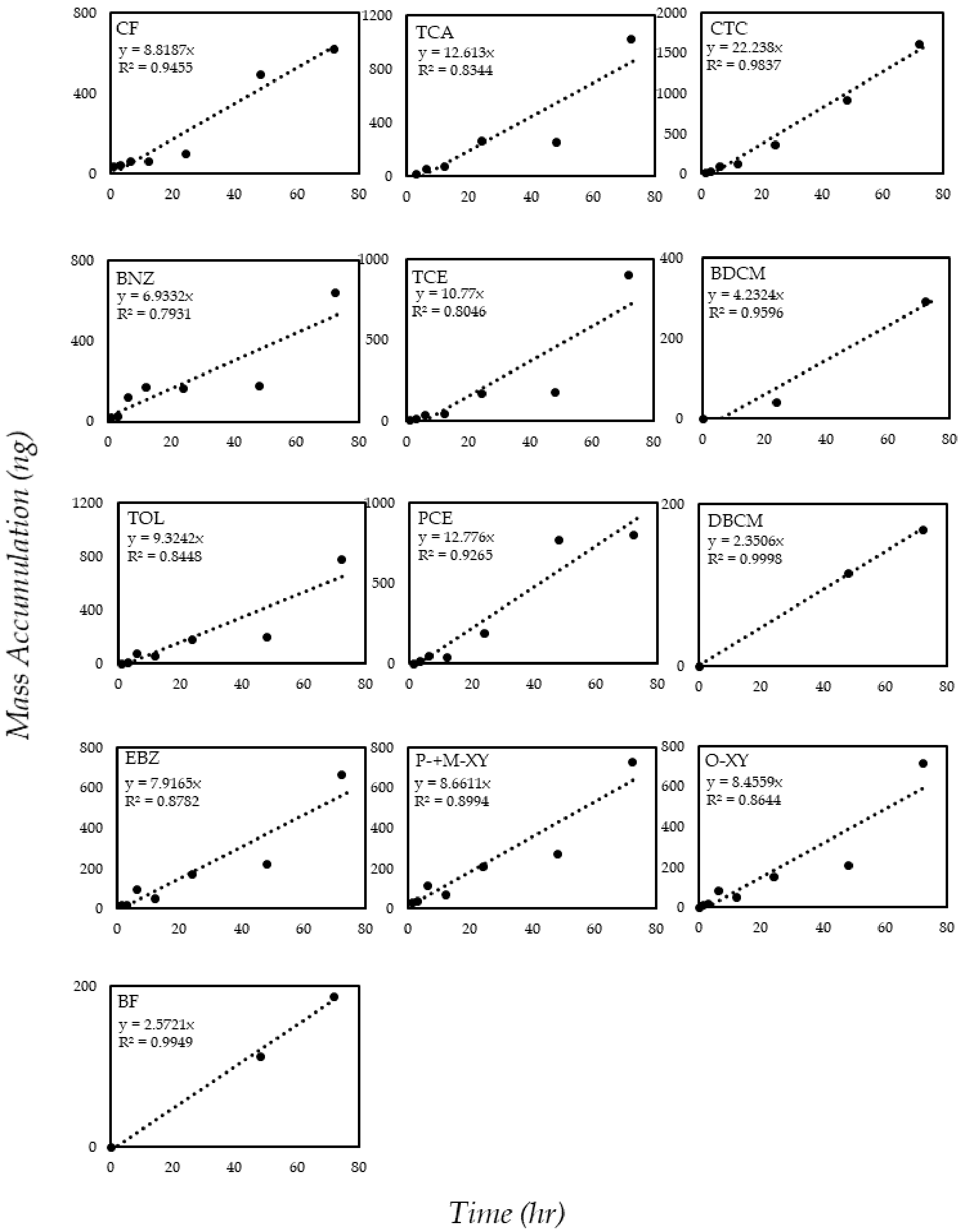

Figure S4. The mass accumulation of VOCs with time, representing the linear kinetics of the solid ceramic dosimeter at a VOC concentration of 43.11 (±6.72) μg/L.

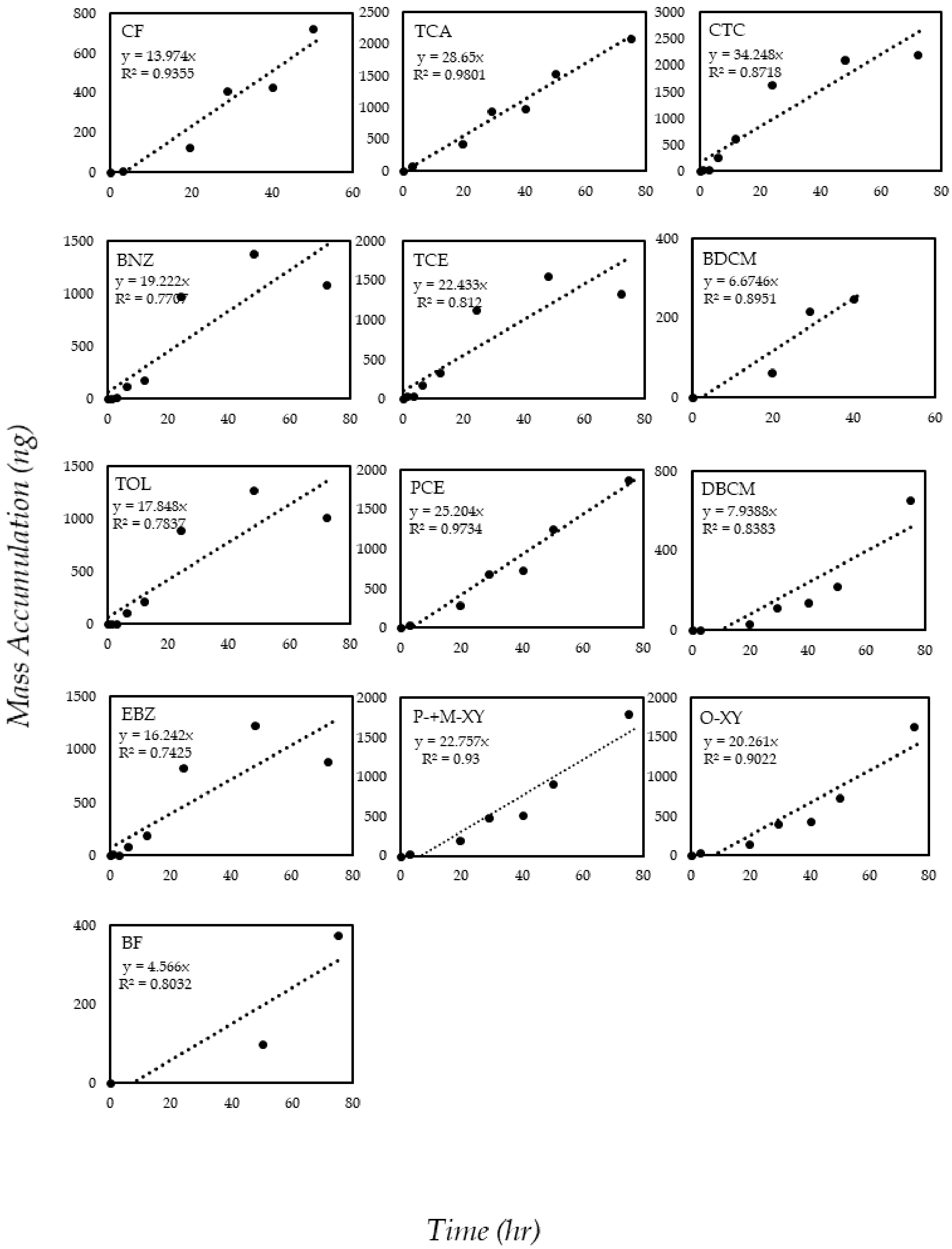

Figure S5. The mass accumulation of VOCs with time, representing the linear kinetics of 81.36 (±15.36) μg/L.

Table S1. Retention time (RT) and SRM transition for VOCs, GC-MS.

Compound	RT (min)	Primary Ion (Da)	GC-MS				
			Spiking Level (ug/L)	MDL (ug/L)	LOQ (ug/L)	RSD (%)	Recovery (%)
**CF**	12.9	83	100	84.17	267.81	25.43	105.32
**TCA**	13.36	97	20	8.9	28.3	13.49	104.89
**CTC**	13.71	117	100	13.27	42.22	4.85	87.07
**BNZ**	14.07	78	50	23.79	75.71	13.35	113.43
**TCE**	15.32	95	100	19.27	61.31	6.43	95.28
**BDCM**	16.42	83	20	9.41	29.93	14.98	99.87
**TOL**	17.83	92	20	8.33	26.5	12.52	105.87
**PCE**	18.98	164	20	9.61	30.59	15.37	99.5
**DBCM**	19.42	129	20	8.78	27.95	14.54	96.1
**EBZ**	20.87	91	20	5.88	18.72	9.36	99.96
**PXY &MXY**	21.12	91	20	5.43	17.28	8.58	100.67
**OXY**	21.94	91	20	7.15	22.75	11.51	98.82
**BF**	22.39	173	20	8.06	25.65	13.37	95.94

© 2020 by the authors. Submitted for possible open access publication under the terms and conditions of the Creative Commons Attribution (CC BY) license (http://creativecommons.org/licenses/by/4.0/).
